# Expansion of regulatory GITR^+^CD25^low/-^CD4^+^ T cells in systemic lupus erythematosus patients

**DOI:** 10.1186/s13075-014-0444-x

**Published:** 2014-09-26

**Authors:** Giuseppe Nocentini, Alessia Alunno, Maria Grazia Petrillo, Onelia Bistoni, Elena Bartoloni, Sara Caterbi, Simona Ronchetti, Graziella Migliorati, Carlo Riccardi, Roberto Gerli

**Affiliations:** Section of Pharmacology, Department of Medicine, University of Perugia, Piazza Severi, Bldg D, 2° floor, I-06132 San Sisto, Perugia, Italy; Rheumatology Unit, Department of Medicine, University of Perugia, Piazza Severi, I-06132 San Sisto, Perugia, Italy

## Abstract

**Introduction:**

CD4^+^CD25^low/-^GITR^+^ T lymphocytes expressing *forkhead box protein P3* (*FoxP3*) and showing regulatory activity have been recently described in healthy donors. The objective of the study was to evaluate the proportion of CD4^+^CD25^low/-^GITR^+^ T lymphocytes within CD4^+^ T cells and compare their phenotypic and functional profile with that of CD4^+^CD25^high^GITR^−^ T lymphocytes in systemic lupus erythematosus (SLE) patients.

**Methods:**

The percentage of CD4^+^CD25^low/-^GITR^+^ cells circulating in the peripheral blood (PB) of 32 patients with SLE and 25 healthy controls was evaluated with flow cytometry. CD4^+^CD25^low/-^GITR^+^ cells were isolated with magnetic separation, and their phenotype was compared with that of CD4^+^CD25^high^GITR^−^ cells. Regulatory activity of both cell subsets was tested in autologous and heterologous co-cultures after purification through a negative sorting strategy.

**Results:**

Results indicated that CD4^+^CD25^low/-^GITR^+^ cells are expanded in the PB of 50% of SLE patients. Expansion was observed only in patients with inactive disease. Phenotypic analysis demonstrated that CD4^+^CD25^low/-^GITR^+^ cells display regulatory T-cell (Treg) markers, including FoxP3, cytotoxic T-lymphocyte-associated protein 4 (CTLA-4), transforming growth factor-beta (TGF-β), and interleukin (IL)-10. In contrast, CD4^+^CD25^high^GITR^−^ cells appear to be activated and express low levels of Treg markers. Functional experiments demonstrated that CD4^+^CD25^low/-^GITR^+^ cells exert a higher inhibitory activity against both autologous and heterologous cells as compared with CD4^+^CD25^high^GITR^−^ cells. Suppression is independent of cell contact and is mediated by IL-10 and TGF-β.

**Conclusions:**

Phenotypic and functional data demonstrate that in SLE patients, CD4^+^CD25^low/-^GITR^+^ cells are fully active Treg cells, possibly representing peripheral Treg (pTreg) that are expanded in patients with inactive disease. These data may suggest a key role of this T-cell subset in the modulation of the abnormal immune response in SLE. Strategies aimed at expanding this Treg subset for therapeutic purpose deserve to be investigated.

## Introduction

Systemic lupus erythematosus (SLE) is an autoimmune disease characterized by loss of tolerance to self-antigens, which is followed by the activation and expansion of autoreactive lymphocytes and the deposition of immune complexes with an inflammatory/necrotic phenomenon in different tissues.

Despite central mechanisms of tolerance, some T cells recognizing self-antigens are released into the periphery, and one of the mechanisms used to control these potentially damaging cells is the activity of regulatory T cells (Tregs). Therefore, it has been suggested that autoimmune diseases derive from the functional or numeric imbalance between autoreactive T cells and Tregs [1-4]. Studies on patients with SLE found that effector T cells are resistant to regulatory activity of Tregs [5,6]. In addition, either reduced number or impaired function of circulating Tregs in SLE has been also reported [7-11]. As an apparent paradox, moreover, some studies described an expansion of Tregs in autoimmune diseases, considered as a homeostatic attempt to control activated effector T cells [12-16]. Thus, conclusive data are still lacking [15,17].

Tregs were originally identified by high surface expression of the CD25 molecule (CD25^high^), but further studies indicated that only CD25^high^ cells expressing the *forkhead box protein P3* (*FoxP3*) transcriptional factor possess regulatory activity [18,19]. Moreover, regulatory functions can be exerted also by CD4^+^CD25^−^ cells [20,21], and in SLE patients, CD4^+^CD25^−^FoxP3^+^ cells with a regulatory activity have been described [14,22].

Other proteins are differentially expressed in Tregs and a number of different Treg subsets, characterized by one or more markers, have been described [23-27]. Among them, GITR is a marker overexpressed in murine CD4^+^ Tregs and with apparently contrasting functions. It favors Treg expansion [28-31], but, in the short term, it inhibits Treg activity by downregulating FoxP3 [32,33]. A problem affecting most Treg markers is that they can be expressed by cells devoid of regulatory activity, such as activated effector cells. For example, the value of CD25 as a Treg marker, particularly in tumor and autoimmune diseases, including SLE, has been questioned after the demonstration that CD25^high^ cells are contaminated by activated effector cells [8,34,35].

Because we recently demonstrated that CD4^+^CD25^low/-^GITR^+^ T cells circulating in healthy subjects possess regulatory activity and other features of Tregs, including anergy [36], we were interested in analyzing number and function of CD4^+^CD25^low/-^GITR^+^ T cells in SLE patients in comparison with those of CD4^+^CD25^high^GITR^−^ cells.

We here demonstrate that CD4^+^CD25^low/-^GITR^+^ cells are expanded in SLE patients, particularly in those with inactive disease. Phenotypic and functional data demonstrate that CD4^+^CD25^low/-^GITR^+^ cells are fully active Tregs, whereas CD4^+^CD25^high^GITR^−^ cells appear to be contaminated by activated effector T cells. Taken together, the present results point to a key role of CD4^+^CD25^low/-^GITR^+^ cells in modulating the immune response in SLE. Strategies focused on the expansion of this Treg subset may offer new therapeutic approaches for SLE treatment.

## Methods

### Study population

Thirty-two patients with SLE, classified according to the American College of Rheumatology criteria [37], were enrolled, and 25 sex- and age-matched subjects acted as healthy controls (HCs). Twenty-five of 32 patients were women, mean age was 44 ± 11 years, and mean disease duration was 9 ± 7 years. Clinical and serologic records were collected at the time of enrollment, and disease activity was measured by using the SLE disease activity index (SLEDAI) [38]. Sixteen patients were taking hydroxycloroquine, 200 mg/day, five patients were receiving mycophenolate mofetil, 2 g/day, and four patients were taking azathioprine, 150 mg/day. Fifteen patients were also receiving less than 5 mg/day of prednisone. The whole study was approved by the local ethics committee “Comitato Etico delle Aziende Sanitarie Regione Umbria” (CEAS), and written informed consent was obtained in accordance with the Declaration of Helsinki.

### Cell isolation

Peripheral blood (PB) mononuclear cells were isolated from heparinized venous blood by gradient separation (Ficoll-Hypaque), and CD4^+^ T cells were magnetically sorted with negative immunoselection by using human CD4^+^ isolation kit II and LD columns (Miltenyi Biotec). Purity of CD4^+^ cells was >98.9%.

To perform real-time polymerase chain reaction (PCR), cell subsets were isolated as previously reported [36]. For CD62L evaluation, CD4^+^CD25^−^GITR^−^ effector T cells (1 × 10^6^ cell/ml) were activated with PMA (50 ng/ml) and ionomycin (250 ng/ml; Sigma-Aldrich) for 3 days.

To perform functional assays, CD4^+^ T cells were passed through MACS columns after incubation with Biotin-anti human CD25 and anti-Biotin microbeads. To avoid contaminating CD25^+^ cells, flow-through cells were passed once again through MACS column (<1% of cells resulted to be CD25^+^), thus obtaining CD25-depleted CD4^+^ T cells (that include effector mixed with CD4^+^CD25^low/-^GITR^+^ cells) [39]. Half of flow-through was stained with PE-anti-human-GITR and subsequently with anti-PE microbeads to obtain CD4^+^CD25^−^GITR^−^ effector cells in the flow-through (cell purity >99%). GITR-depleted CD4^+^ cell population (that include effector and CD4^+^CD25^high^GITR^−^ cells) was obtained similarly, by using anti-GITR Ab instead of anti-CD25 Ab [39].

### Flow cytometry

To evaluate CD4^+^CD25^low/-^GITR^+^ cell percentage, CD4^+^ T cells were stained with PE-anti-human GITR (Biolegend), Biotin-anti-human CD25 followed by anti-Biotin FITC (Miltenyi Biotec), and their respective isotypes [36].

To perform a phenotypical characterization of CD4^+^CD25^low/-^GITR^+^, cells were stained with the following antibodies: PECy7-anti-human CD4 (BD Pharmingen), AF647-anti-human CD25 (AbD Serotec), AF647-anti-human GITR (Biolegend), FITC-anti-human CD45RO (BD Pharmingen), FITC-anti-human CD45RA (BD Pharmingen), and FITC-anti-human CD127 (BD Pharmingen).

For intracellular FOXP3 staining, cells were permeabilized, fixed, and stained with anti-human FOXP3-AF647 (BD Pharmingen) by following the manufacturer’s instructions. To perform CTLA-4 staining, cells were cultured with PMA, ionomycin, and monensin for 4 hours at 37°C, permeabilized with saponin buffer, and incubated with PE-anti-human CTLA-4 (BD Pharmingen) [36].

Samples were analyzed by using Beckman Coulter EPICS XL-MCL flow cytometer running EXPO32 ADC analysis software and BD Facscalibur running BD CellQuest Pro analysis software.

### Real-time PCR

Total RNA was isolated with the RNeasy Mini extraction kit (Qiagen), and RNA was reverse transcribed with Quantitect reverse transcription kit (Qiagen). Real-time PCR was performed in a Chromo 4 Four-Color Real-Time System (Bio-Rad) using Taqman probes (Applied Biosystems). The VIC-labeled housekeeping gene, *hypoxanthine phosphoribosyl transferase 1* (*HPRT*)*-1*, was amplified in the same tube of the FAM-labeled investigated gene (four replicates). The ΔΔCt method was used to determine expression of the gene of interest, and the expression of effector cells were set equal to 1 [39].

### Functional assays

Effector T cells were stained with CFSE (Invitrogen, Life Technologies); 2 μ*M*) and co-cultured with unlabeled effector, CD25-depleted or GITR-depleted CD4^+^ cells in supplemented RPMI medium (0.25 × 10^5^ and 0.75 × 10^5^ cells/well, respectively; 5 × 10^5^ cells/ml). Where indicated, neutralizing anti-IL-10 (30 ng/ml; BD Pharmingen), anti-TGF-β (0.5 μg/ml; R&D Systems) or isotype controls were added.

Transwell experiments were performed in 24-well Transwell plates with a 0.4 mm pore size (Corning). TCR activation was obtained by coating the bottom and the top chambers of the Transwell plates by cross-linked goat anti-mouse mAb (Southern Biotech) and mouse anti-human CD3ε [36]. CFSE-labeled effectors were seeded in the bottom chamber of the 24-well plate at a concentration of 0.5 × 10^5^ cells/well. The unlabeled cells (effectors, CD25-depleted or GITR-depleted CD4^+^ cells) were plated in the top chamber of the transwell at 1.5 × 10^5^ cells/well.

CFSE dilution was evaluated after 4-day culture with FACS analysis after TCR stimulation [36]. Regulatory activity of CD4^+^CD25^low/-^GITR^+^ and CD4^+^CD25^high^GITR^−^ cells was calculated by comparing cell proliferation rate of effector cells co-cultured with effector cells with that of effectors co-cultured with CD25-depleted or GITR-depleted CD4^+^ cells, respectively, as previously described [36].

### Statistical analysis

Mann Whitney *U* test was used to compare HC and SLE patients. Kruskal-Wallis test with Dunn’s test for multiple comparison *post hoc* were used for the comparison between HC, active SLE, and inactive SLE. Spearman correlation coefficient, χ^2^ test, and binary logistic regression were also used, as detailed throughout the manuscript. The significance level was two-sided and set at *P* <0.05. All data analysis was performed by using IBM-SPSS version 13.0.

## Results

### CD4^+^CD25^low/-^GITR^+^ T cells are expanded in the PB of SLE patients and particularly in those with inactive disease

**Figure 1:**
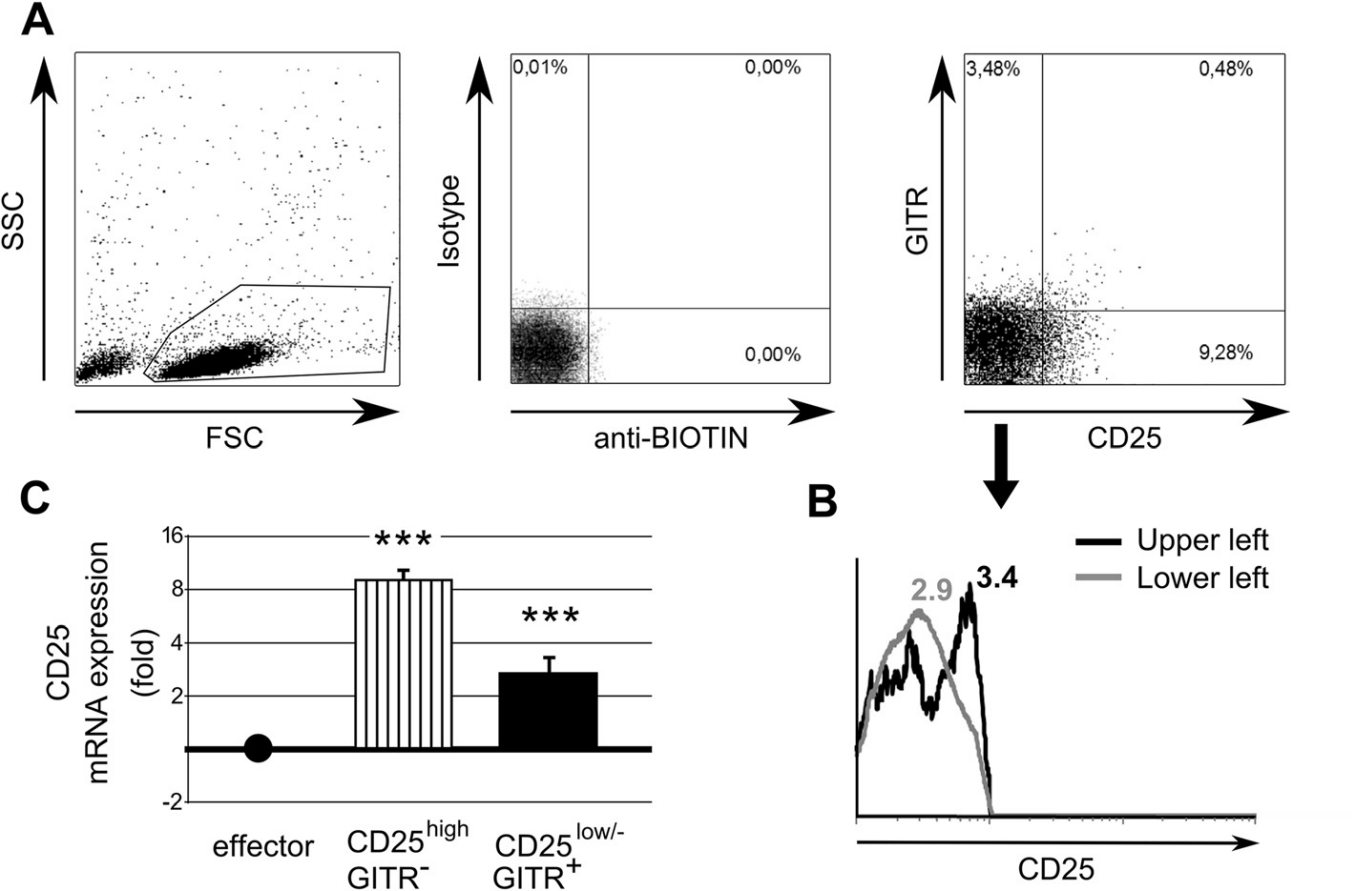
**Detection of circulating CD4** ^**+**^ **CD25** ^**low/-**^ **GITR** ^**+**^ **cells in SLE patients** ***.*** **(A)** Flow-cytometry analysis of anti-GITR and anti-CD25-stained CD4^+^ T lymphocytes, freshly isolated from blood of a representative SLE patient, reveals the presence of a CD25^−^GITR^+^ cell subpopulation. **Left panel:** gating strategy. Gated CD4^+^ T lymphocytes were stained with isotype control Abs **(middle panel)** or anti-GITR and anti-CD25 Abs **(right panel)**. **(B)** the levels of CD25 expression were evaluated in the upper left and lower left quadrants and MFI is reported. **(C)** mRNA expression of *CD25* (FAM6 fluorochrome) in the indicated subpopulations was evaluated in quadruplicate with real-time PCR. In the same tube, expression of the housekeeping gene *HPRT1* (VIC fluorochrome) was evaluated for normalization. Values of *CD25* expression (striped column, CD25^high^GITR^−^; black column, CD25^low/-^GITR^+^) is shown as fold increase of mRNA levels in the positively sorted subpopulations over mRNA levels in effector (CD4^+^CD25^−^GITR^−^) T cells, arbitrarily set equal to 1. Data shown are mean ± SD of four SLE patients. ***P <0.001.

**Figure 2:**
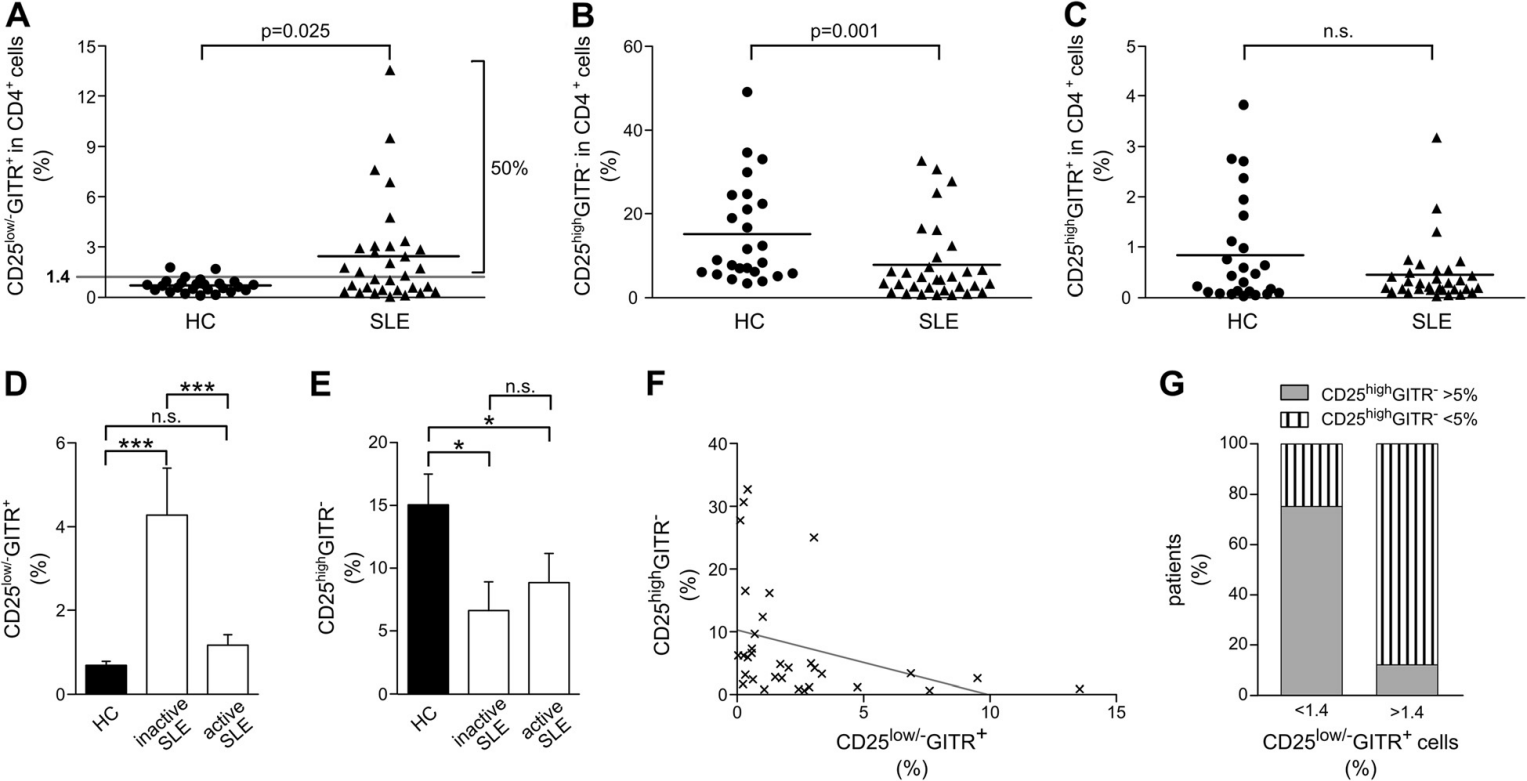
**Percentages of circulating CD4** ^**+**^ **CD25** ^**low/-**^ **GITR** ^**+**^ **and CD4** ^**+**^ **CD25** ^**high**^ **GITR** ^**−**^ **cells in HC are different from those in SLE patients** ***.*** Percentage of CD4^+^CD25^low/-^GITR^+^ **(A)**, CD4^+^CD25^high^GITR^−^ **(B)** and CD4^+^CD25^high^GITR^+^ **(C)** in CD4^+^ T lymphocytes evaluated with flow-cytometry analysis of anti-GITR- and anti-CD25-stained CD4^+^ T lymphocytes, isolated from PB of 25 HC and 32 SLE patients, is shown. Horizontal lines indicate mean percentage. *P* values are according to Mann-Whitney *U* test comparing differences in HC and SLE. The percentage of patients with more than 1.4% CD4^+^CD25^low/-^GITR^+^ cells (90th percentile of HC) is also indicated **(A)**. Percentage of CD4^+^CD25^low/-^GITR^+^ **(D)** and CD4^+^CD25^high^GITR^−^ **(E)** in CD4^+^ T cells purified from freshly isolated PB of HC, patients with inactive disease identified by an SLEDAI =0 (*n* =13), and patients with active disease identified by an SLEDAI >0 (*n* =19) was evaluated. Bars indicate mean ± SEM. n.s., *P* >0.05, *P <0.05 and ***P <0.001, according to Kruskal-Wallis test comparing active SLE, inactive SLE, and HC. **(F)** Correlation between levels of CD4^+^CD25^low/-^GITR^+^ and CD4^+^CD25^high^GITR^−^ in CD4^+^ cells purified from freshly isolated SLE patients (Spearman ρ = −0.5; *P* <0.01). **(G)** Distribution of CD4^+^CD25^high^GITR^−^ cells according to the expansion of CD4^+^CD25^low/-^GITR^+^ cells. SLE patients with a percentage of these cells higher than 1.4% (90th percentile of the distribution in HC) were defined as having expansion of the CD4^+^CD25^low/-^GITR^+^ cells (16 of 32 patients). *P* <0.001 according to χ^2^ test on raw data.

The percentage of CD4^+^ Tregs characterized by *GITR* expression and low/negative levels of CD25 (CD4^+^CD25^low/-^GITR^+^), recently described in HC [36], was evaluated in SLE patients with flow cytometry (Figure 1A). As in HC [36], the levels of expression of *CD25* in this subset (formally CD25^−^) were higher than those in CD25^−^GITR^−^ cells (effectors), as shown by both real-time PCR and flow cytometry (Figure 1B,C). SLE patients with a percentage of CD4^+^CD25^low/-^GITR^+^ cells higher than 1.4% (90th percentile of the distribution in HCs) were defined as having an expansion of CD4^+^CD25^low/-^GITR^+^ cells (number 16; 50%). Consequently, the mean value of circulating CD4^+^CD25^low/-^GITR^+^ Tregs in SLE was significantly higher than detected in HCs (Figure 2A). This result was in striking contrast with that observed in CD4^+^CD25^high^GITR^−^ and CD4^+^CD25^high^GITR^+^ Tregs, which were in lower proportion and equal in SLE patients, respectively (Figure 2B,C).

Taking into account the wide range of expansion of circulating CD4^+^CD25^low/-^GITR^+^ cells in SLE, we wondered whether they could be somehow related to general disease activity. To this purpose, patients were divided into two groups according to SLEDAI score: inactive disease patients with SLEDAI =0 (*n* =13) and active-disease patients with SLEDAI >0 (*n* =19). As shown in Figure 2D, inactive patients had a percentage of PB CD4^+^CD25^low/-^GITR^+^ cells higher than those in active patients, whereas the CD4^+^CD25^high^GITR^−^ Treg percentage was low, irrespective of disease activity (Figure 2E). Spearman correlation coefficient or binary logistic regression was used to identify a possible relation between CD4^+^CD25^low/-^GITR^+^ percentages and other clinical variables such as age or therapy, but no significant difference was observed.

Interestingly, an inverse correlation was found between CD4^+^CD25^low/-^GITR^+^ and CD4^+^CD25^high^GITR^−^ cell percentage (Figure 2F). In particular, 15 of 16 patients showing a CD4^+^CD25^high^GITR^−^ percentage <5% had a CD4^+^CD25^low/-^GITR^+^ percentage higher than 1.4%, and 12 of 16 patients showing a CD4^+^CD25^high^GITR^−^ percentage higher than 5% had a CD4^+^CD25^low/-^GITR^+^ percentage lower than 1.4% (Figure 2G).

### CD4^+^CD25^low/-^GITR^+^ but not CD4^+^CD25^high^GITR^−^ cells show the same phenotype in SLE as in HC

Because circulating activated T cells are found in autoimmune disorders, and CD25 and GITR are also markers of activated effector T cells [27,40-42], we performed a phenotypic characterization of CD4^+^CD25^low/-^GITR^+^ and CD4^+^CD25^high^GITR^−^ cells in SLE patients to verify whether they showed a Treg or activated phenotype. The phenotype of each cell population was compared to that of effector CD4^+^ T cells (CD4^+^CD25^−^GITR^−^) and the respective cell populations from HC.

**Figure 3:**
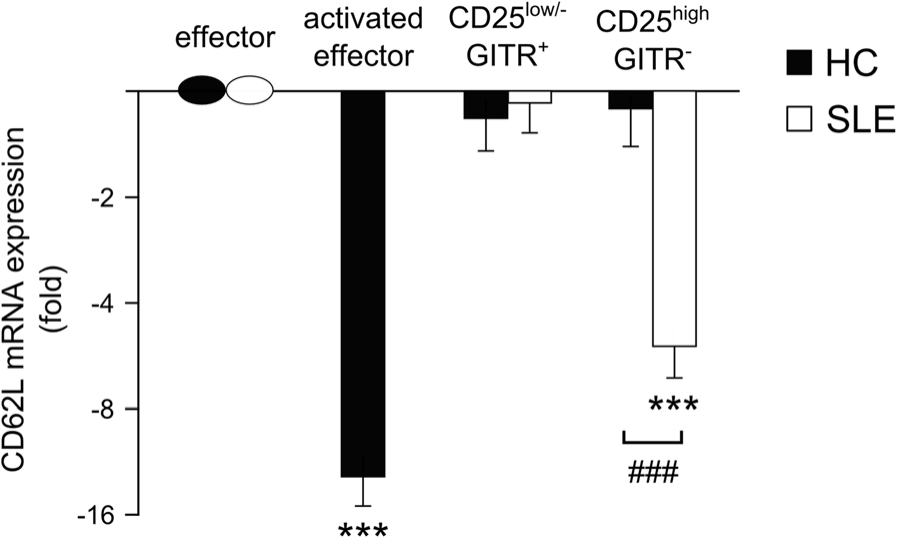
**CD4** ^**+**^ **CD25** ^**low/-**^ **GITR** ^**+**^ **cells from HC and SLE patients express comparable levels of CD62L** ***.*** CD62L mRNA expression (fold decrease expressed in a base 2 logarithmic scale) in CD4^+^CD25^low/-^GITR^+^ and CD4^+^CD25^high^GITR^−^ T cells over the respective naïve effector CD4^+^CD25^−^GITR^−^ cell population, arbitrarily set equal to 1. The levels of mRNA expression in effectors from HC and SLE patients were similar. Expression level of CD62L mRNA in CD4^+^CD25^−^GITR^−^ effector cells activated by 72-hour treatment with PMA plus ionomycin is also reported as control. Bars indicate mean ± SEM. ***P <0.001, according to Mann Whitney *U* test comparing expression in Treg cell population (CD4^+^CD25^low/-^GITR^+^ and CD4^+^CD25^high^GITR^−^) with that of the respective CD4^+^CD25^−^GITR^−^ cell population. ^###^ *P* <0.001, according to Mann-Whitney *U* test comparing expression in HC and SLE.

*CD62L* expression is known to be high in naïve and memory CD4^+^ effector cells and low in activated cells, and CD4^+^CD25^+^CD62L^+^ but not CD4^+^CD25^+^CD62L^−^ cells have been found to possess regulatory activity [43,44]. Figure 3 shows that in HC, the mRNA levels of CD62L were comparable in untreated naïve effector, CD4^+^CD25^low/-^GITR^+^ and CD4^+^CD25^high^GITR^−^ cells and much lower (about ten fold) in PHA/ionophore-activated effectors. In SLE patients, the mRNA levels of CD62L were comparable in effector and CD4^+^CD25^low/-^GITR^+^ cells, suggesting that these cells were not activated effector cells and possibly were Treg cells maintaining regulatory activity. Conversely, CD4^+^CD25^high^GITR^−^ cells showed much lower levels of CD62L, thereby suggesting that this subset includes activated effector T cells or Treg cells devoid of regulatory activity.

**Figure 4:**
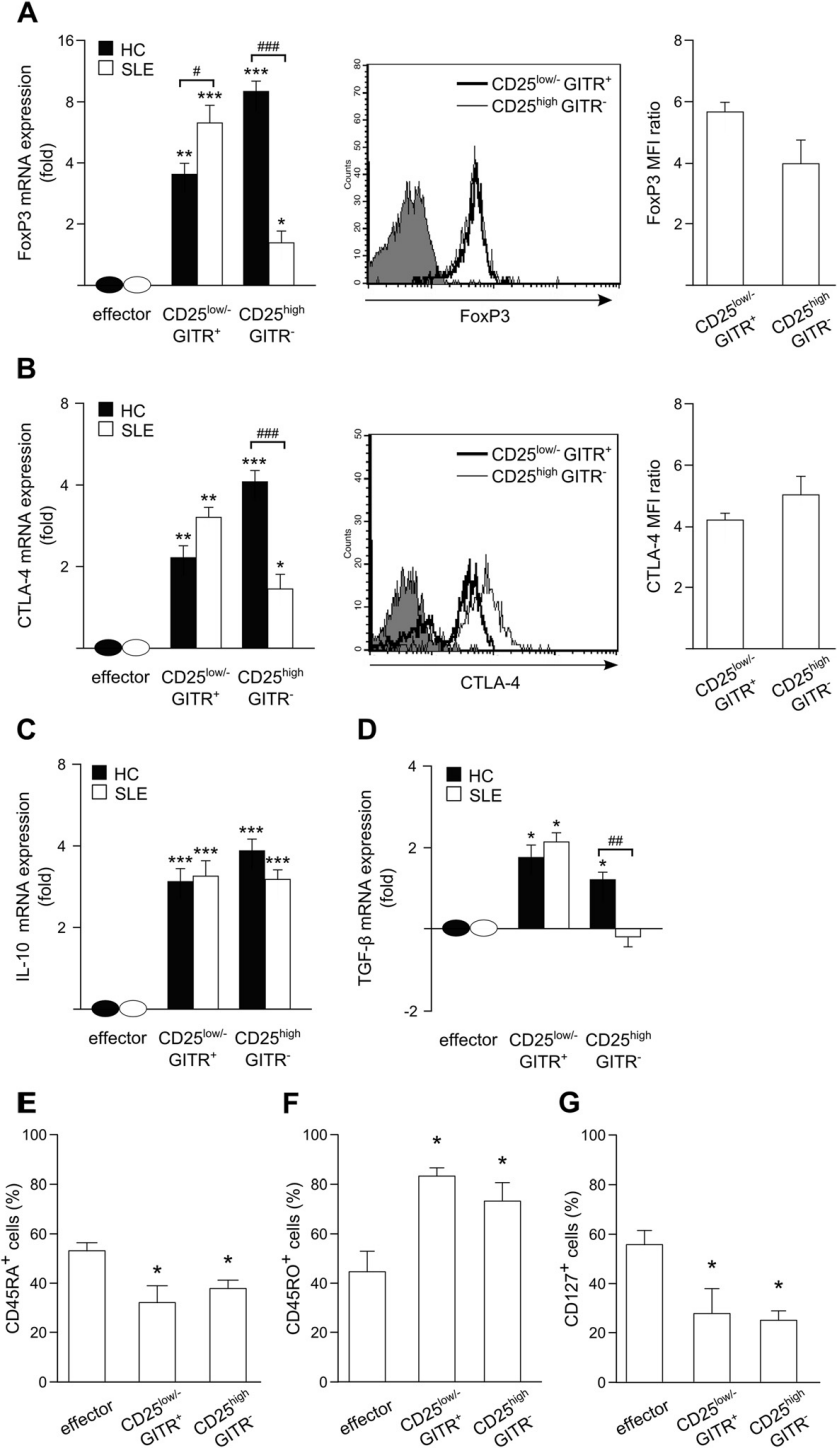
**CD4** ^**+**^ **CD25** ^**low/-**^ **GITR** ^**+**^ **cells from HC and SLE patients express comparable levels of Treg markers** ***.*** Treg-marker mRNA expression (fold increase expressed in a base 2 logarithmic scale) in CD4^+^CD25^low/-^GITR^+^ and CD4^+^CD25^high^GITR^−^ T cells from HC and SLE patients over the respective effector CD4^+^CD25^−^GITR^−^ cell population arbitrarily set equal to 1 **(A**, **B**, **left** **C**, **D)**. The levels of mRNA expression of genes in effectors from HC and SLE patients were similar. Bars indicate mean ± SEM. *P <0.05; **P <0.01’ and ***P <0.001, according to Mann-Whitney *U* test comparing expression in Treg cell population (CD4^+^CD25^low/-^GITR^+^ and CD4^+^CD25^high^GITR^−^) with the respective CD4^+^CD25^−^GITR^−^ cell population. ^#^ *P* <0.05; ^##^ *P* <0.01; and ^###^ *P* <0.001, according to Mann-Whitney *U* test comparing expression in HC and SLE. **(A**, **B**, **middle** and **right)** Expression of FoxP3 **(A)** and CTLA-4 **(B)** in the indicated subpopulations was evaluated with flow cytometry. For CTLA-4 evaluation, cells were treated with 4 hours PMA plus ionomycin and monensin. Solid histogram represents isotype control. Results obtained with T cells from a representative SLE patient are shown. **(A**, **B right)** MFI ratios (MFI of Ab-stained cells/MFI of isotype-stained cells) are mean ± SEM (bars) of three SLE patients. *P >0.05, according to Mann-Whitney *U* test comparing expression in CD4^+^CD25^low/-^GITR^+^ cell population with that in CD4^+^CD25^high^GITR^−^ cell population. **(E-G)** Expression of CD45RO, CD45RA, and CD127 in the indicated subpopulations was evaluated with flow cytometry. Percentages of positive cells are mean ± SEM (bars) of three SLE patients. *P <0.05, according to Mann-Whitney *U* test comparing expression in Treg cell population (CD4^+^CD25^low/-^GITR^+^ and CD4^+^CD25^high^GITR^−^) with that of CD4^+^CD25^−^GITR^−^ cell population.

We next evaluated the expression of the main Treg markers. In CD4^+^CD25^low/-^GITR^+^ cells from SLE patients, the mRNA expression of CTLA-4, IL-10, and TGF-β was similar to those seen in CD4^+^CD25^low/-^GITR^+^ cells from HC, and the mRNA expression of FoxP3 was even higher (Figure 4A through D). At the protein level, the expression of *FoxP3* and *CTLA-4* was similar to those seen in CD4^+^CD25^low/-^GITR^+^ cells from HC [36], demonstrating that CD4^+^CD25^low/-^GITR^+^ cells from SLE patients display a Treg phenotype, as demonstrated in HC (Figure 4A, B).

Conversely, the level of FoxP3 mRNA in CD4^+^CD25^high^GITR^+^ cells was lower than in CD4^+^CD25^low/-^GITR^+^ cells, whereas the expression of FoxP3 protein was similar (Figure 4A). Such a result is relevant because the expression of *FoxP3* in HC is much higher in CD4^+^CD25^high^GITR^+^ cells than in CD4^+^CD25^low/-^GITR^+^ cells [36]. The expression of *CTLA-4* was lower in CD4^+^CD25^high^GITR^+^ cells than in CD4^+^CD25^low/-^GITR^+^ cells at the mRNA level while being similar at the protein level (Figure 4B). Even in this case, such a result is relevant because the expression of *CTLA-4* in HC is as much as threefold higher in CD4^+^CD25^high^GITR^+^ cells than in CD4^+^CD25^low/-^GITR^+^ cells [36]. Moreover, TGF-β was not expressed in CD4^+^CD25^high^GITR^−^ cells from SLE patients.

Overall, our data suggest that most of the cells belonging to CD4^+^CD25^high^GITR^−^ subset and circulating in SLE patients does not display a Treg phenotype.

The levels of *CD45RO* and *CD45RA* expression found in CD4^+^CD25^low/-^GITR^+^ and CD4^+^CD25^high^GITR^+^ cells (Figure 4E,F) suggest that the memory phenotype of both subsets is more predominant in SLE patients than in HC [36]. Interestingly, *CD127* expression levels in CD4^+^CD25^low/-^GITR^+^ cells (Figure 4G) were lower than those observed in HC [36].

### In SLE, CD4^+^CD25^low/-^GITR^+^ cells show a normal regulatory activity, whereas CD4^+^CD25^high^GITR^−^ cells show a weak regulatory activity

Next, we verified the *in vitro* functional activity of CD4^+^CD25^low/-^GITR^+^ and CD4^+^CD25^high^GITR^−^ cells from SLE patients. Because anti-GITR Ab must be used to isolate GITR^+^ cells and GITR binding inactivates Treg activity [29,36,45,46], isolated GITR^+^ cells cannot be used to evaluate their regulatory activity. Therefore, we adopted a previously described culture system in which CFSE-labeled effectors (CD4^+^CD25^−^GITR^−^), obtained by depletion of both CD25^+^ and GITR^+^ cells, are co-cultured with CD25^+^ cell-depleted CD4^+^ cells, containing physiological levels of CD4^+^CD25^low/-^GITR^+^ cells (CD25^−^GITR^−^ plus CD25^low/-^GITR^+^) [36,39,47]. To test their regulatory activity, the proliferation of CFSE-labeled effectors co-cultured with effectors and CFSE-labeled effectors co-cultured with “CD25^−^GITR^−^ plus CD25^low/-^GITR^+^” cells were compared. A similar approach was used to test the regulatory activity of CD4^+^CD25^high^GITR^−^ cells. In particular, the proliferation of CFSE-labeled effectors co-cultured with effectors and CFSE-labeled effectors co-cultured with GITR^+^ cell-depleted CD4^+^ cells (CD25^−^GITR^−^ plus CD25^high^GITR^−^) were compared.

**Figure 5:**
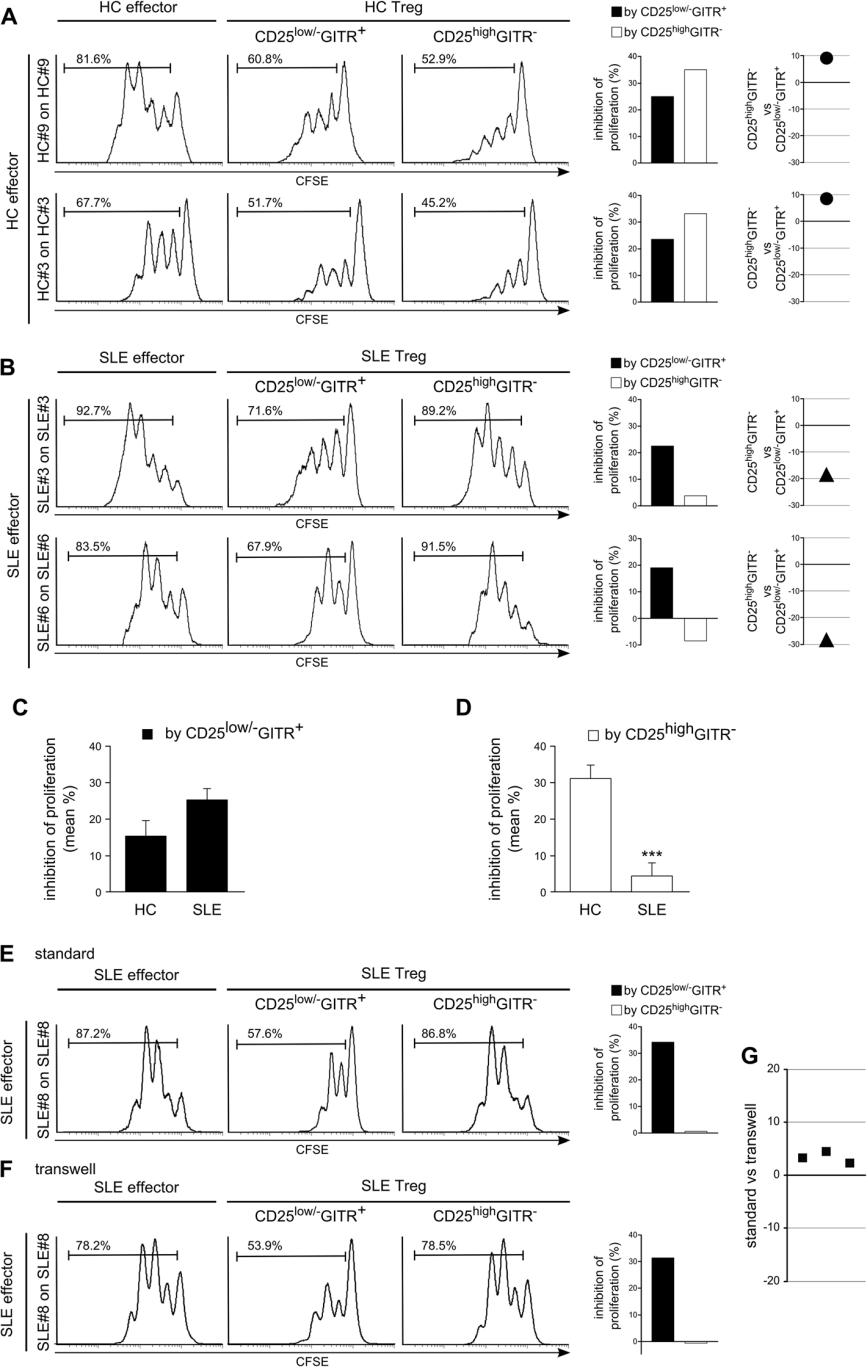
**CD4** ^**+**^ **CD25** ^**low/-**^ **GITR** ^**+**^ **but not CD4** ^**+**^ **CD25** ^**high**^ **GITR** ^**−**^ **cells from SLE patients exert a regulatory activity similar to HC.** Regulatory activity is independent of cell contact*.* CFSE-labeled effectors, activated with cross-linked anti-CD3 Ab, were co-cultured with autologous unlabeled effectors (CD4^+^CD25^−^GITR^−^) **(left, A, B)**, unlabeled CD25-depleted (including CD4^+^CD25^−^GITR^−^ and CD4^+^CD25^low/-^GITR^+^) **(middle, A, B)** or unlabeled GITR-depleted (including CD4^+^CD25^−^GITR^−^ and CD4^+^CD25^high^GITR^−^) cells **(right, A, B)** at 1:3 cell ratio. Proliferation was evaluated after 4 days with flow cytometry. Graphs show inhibition of proliferation by CD4^+^CD25^low/-^GITR^+^ and CD4^+^CD25^high^GITR^−^ **(**histograms **A, B)** and the differences between the inhibition (plot on the right) in each subject **(A, B)**. Experiments with cells from PBs of two representative HCs **(A)** and two representative SLE patients **(B)** are shown. **(C, D)** Histograms show mean percentage of inhibition of proliferation by CD4^+^CD25^low/-^GITR^+^ **(C)** and CD4^+^CD25^high^GITR^−^ **(D)** cells from twelve HCs and six SLE patients. **(E, F)** Inhibition by CD4^+^CD25^low/-^GITR^+^ and CD4^+^CD25^high^GITR^−^ in an identical experiment as the described (classical) and in transwell experiments (unlabeled cells above and CFSE-labeled cells below the transwell). A representative SLE patient of 3 is shown. Graph in **G** shows differences in the inhibition of proliferation by CD4^+^CD25^low/-^GITR^+^ in the classic and transwell experiments in the three patients. ***P <0.001 according to Mann-Whitney *U* test, comparing inhibition by CD4^+^CD25^high^GITR^−^ cells from HC and SLE patients.

Our data demonstrated that CD4^+^CD25^low/-^GITR^+^ cells from SLE patients exert regulatory activity on the proliferation of the respective autologous effectors at levels as similar as those seen in HC (Figure 5A-C), confirming that they are Tregs. Figure 5C shows that the mean regulatory activity by CD4^+^CD25^low/-^GITR^+^ cells from SLE patients was higher than that exerted by the same cells from HC, but the difference was not significant. Not always did CD4^+^CD25^high^GITR^−^ cells from SLE patients exert a regulatory activity on the proliferation of the respective autologous effectors (Figure 5B), and their regulatory activity was significantly lower than in HCs (Figure 5D), confirming functional differences between this subset in SLE patients and HCs.

**Figure 6:**
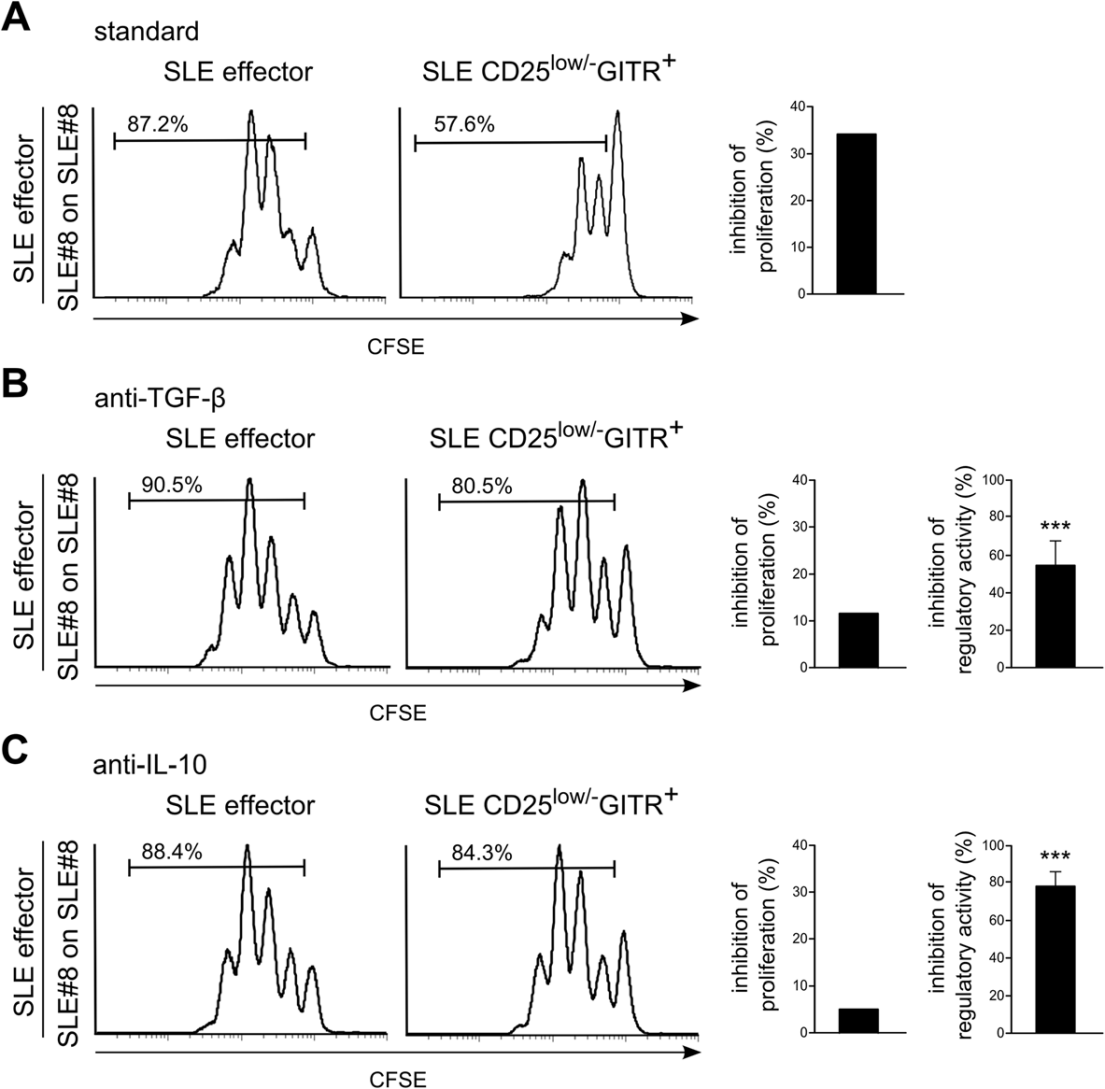
**Blocking TGF-β and IL-10 inhibits the regulatory activity of CD4** ^**+**^ **CD25** ^**low/-**^ **GITR** ^**+**^ **cells from SLE patients** ***.*** **(A-C)** CFSE-labeled effectors, activated with cross-linked anti-CD3 Ab, were co-cultured with autologous unlabeled effectors (CD4^+^CD25^−^GITR^−^) **(left)** or unlabeled CD25-depleted (including CD4^+^CD25^−^GITR^−^ and CD4^+^CD25^low/-^GITR^+^) **(right)** at a 1:3 cell ratio. In panel **B**, blocking anti-TGF-β Ab (0.5 μg/ml) and, in panel **C**, blocking anti-IL-10 Ab (30 ng/ml) were added. Proliferation was evaluated after 4 days with flow cytometry. Histograms on the left show inhibition of proliferation by CD4^+^CD25^low/-^GITR^+^ in the presence **(B, C)** or absence **(A)** of blocking Abs. One representative SLE patient is shown. Histograms on the right show the mean inhibitory effect of blocking anti-TGF-β Ab **(B)** and anti-IL-10 Ab **(C)** on the regulatory activity of CD4^+^CD25^low/-^GITR^+^ cells, observed in cells from three SLE patients ± SEM (bars). **P <0.01 according to the Mann-Whitney *U* test.

To evaluate the mechanism by which CD4^+^CD25^low/-^GITR^+^ cells exert their regulatory activity, we performed experiments by using transwells. Figures 5E-G shows that the regulatory activity of CD4^+^CD25^low/-^GITR^+^ cells is comparable with or without transwells, demonstrating that the regulatory activity of CD4^+^CD25^low/-^GITR^+^ cells is exerted without cell contact and suggesting that regulatory activity relies on soluble factors. Indeed, blocking Abs directed against TGF-β and IL-10 inhibited the regulatory activity of CD4^+^CD25^low/-^GITR^+^ cells partially or completely, demonstrating that both cytokines mediate the regulatory activity (Figure 6).

**Figure 7:**
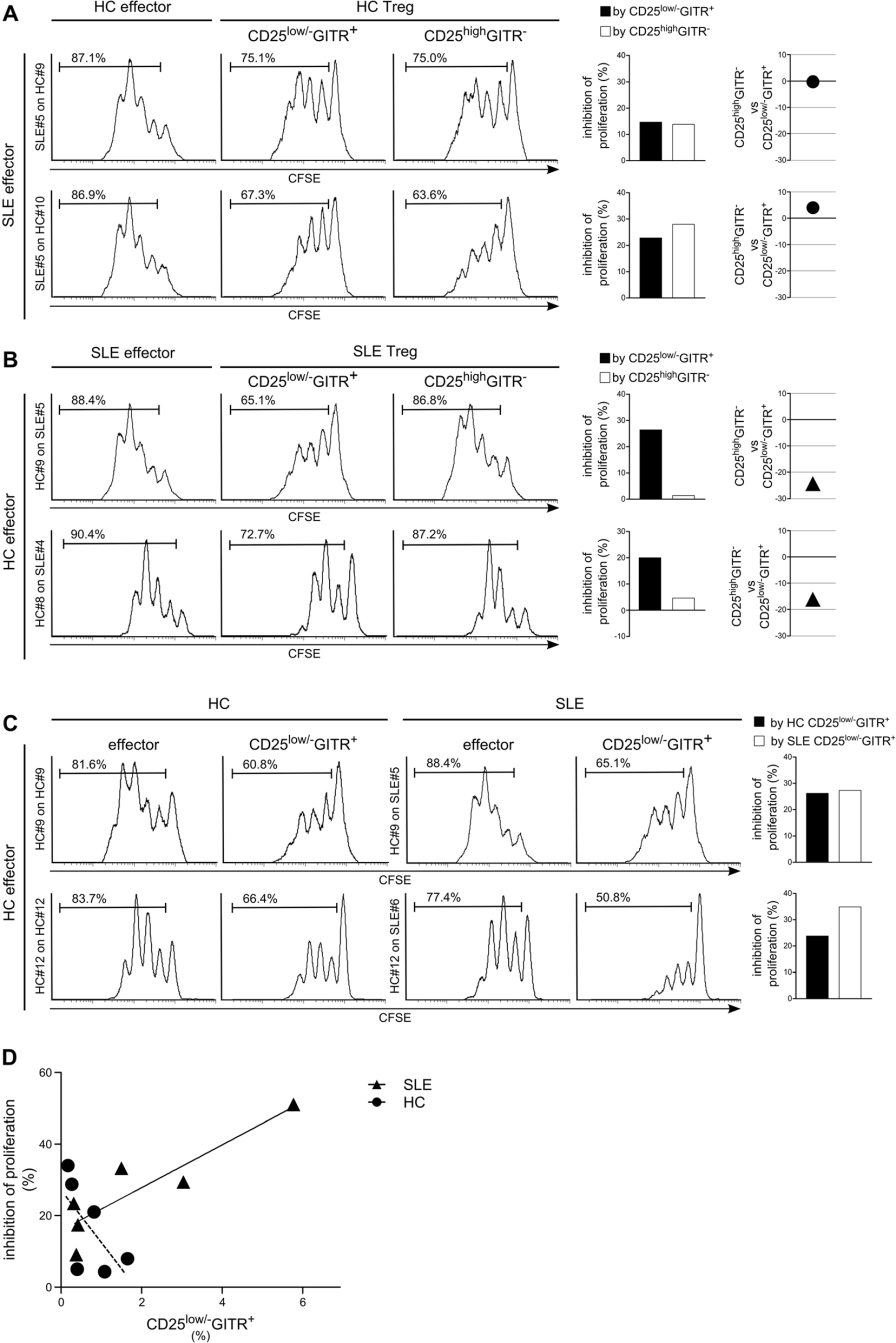
**In SLE patients, the regulatory activity exerted by CD4** ^**+**^ **CD25** ^**low/-**^ **GITR** ^**+**^ **cells is higher than CD4** ^**+**^ **CD25** ^**high**^ **GITR** ^**−**^ **cells** ***.*** CFSE-labeled effector T cells from SLE **(A)** or HC **(B)** patients, activated with cross-linked anti-CD3 Ab, were co-cultured with heterologous unlabeled effector (CD4^+^CD25^−^GITR^−^) **(left, A** and **B)**, heterologous unlabeled CD25-depleted (including CD4^+^CD25^−^GITR^−^ and CD4^+^CD25^low/-^GITR^+^) **(middle**, **A** and **B)** or heterologous unlabeled GITR-depleted (including CD4^+^CD25^−^GITR^−^ and CD4^+^CD25^high^GITR^−^) cells **(right, A** and **B)** at a 1:3 cell ratio from HC **(A)** or SLE patients **(B)**. Proliferation was evaluated after 4 days with flow cytometry. Experiments with cells from two representative SLE patients co-cultured with cells from two representative HCs are shown. Graphs show inhibition of proliferation by CD4^+^CD25^low/-^GITR^+^ and CD4^+^CD25^high^GITR^−^ (histograms) and the differences between the inhibition (plots) in each subject **(A, B)**. **(C)** CFSE-labeled effector T cells from HC, activated with cross-linked anti-CD3 Ab, were co-cultured with HC **(left)** or SLE **(middle right)** unlabeled effector (CD4^+^CD25^−^GITR^−^) or HC **(middle left)** or SLE **(right)** unlabeled CD25-depleted (including CD4^+^CD25^−^GITR^−^ and CD4^+^CD25^low/-^GITR^+^) cells at a 1:3 cell ratio. Histogram show inhibition of proliferation of labeled HC effector by HC and SLE CD4^+^CD25^low/-^GITR^+^ cells. **(D)** Correlation between levels of CD4^+^CD25^low/-^GITR^+^ in CD4^+^ cells purified from HC and SLE patients and inhibition of proliferation by CD4^+^CD25^low/-^GITR^+^ cells from the same subjects (CD4^+^CD25^low/-^GITR^+^ cells from SLE patients; Spearman ρ =0.77, *P* =0.10; CD4^+^CD25^low/-^GITR^+^ cells from HC, Spearman ρ = −0.71, *P* =0.13).

**Figure 8:**
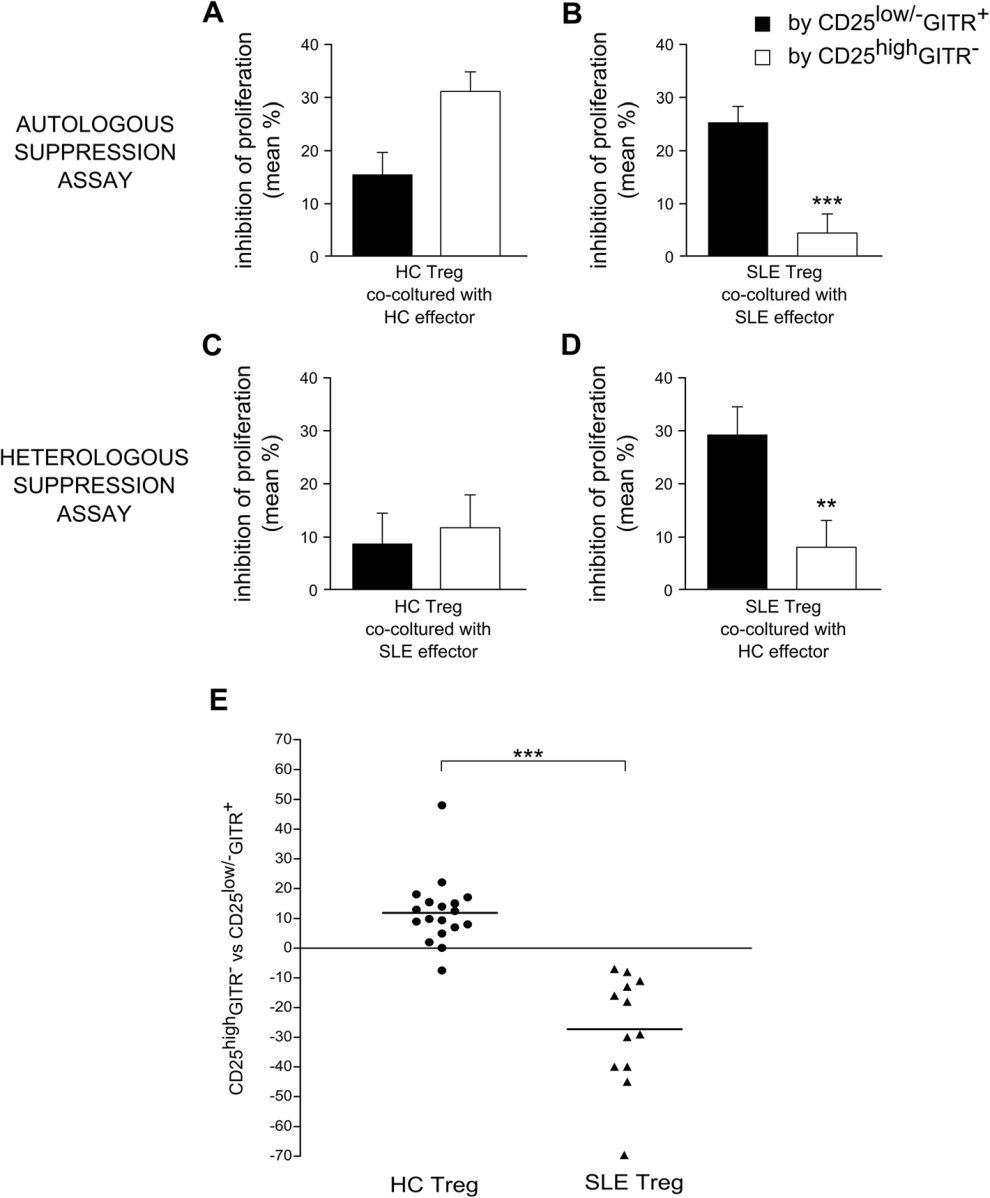
**Regulatory activity of CD4** ^**+**^ **CD25** ^**low/-**^ **GITR** ^**+**^ **and CD4** ^**+**^ **CD25** ^**high**^ **GITR** ^**−**^ **cells from HC differs from that of SLE patients** ***.*** Inhibition of proliferation by CD4^+^CD25^low/-^GITR^+^ cells and CD4^+^CD25^high^GITR^−^ cells observed in autologous (see Figure 5) and heterologous (see Figure 7) co-cultures is shown **(A-D)**. **P <0.01 and ***P <0.001 according to Mann-Whitney *U* test comparing inhibition by CD4^+^CD25^low/-^GITR^+^ cells and CD4^+^CD25^high^GITR^−^ cells. **(E)** Differences between regulatory activity exerted by CD4^+^CD25^high^GITR^−^ and CD4^+^CD25^low/-^GITR^+^ Treg from each HC (circle) and SLE patient (triangle) is reported, as shown in right plots of Figures 5 and 7. Horizontal lines indicate mean differences. ***P <0.001, according to the Mann-Whitney *U* test comparing differences in HC and SLE.

We next tested the effects of CD4^+^CD25^low/-^GITR^+^ and CD4^+^CD25^high^GITR^−^ cells from SLE patients over heterologous effectors from HC (Figure 7B). As control, the effects of CD4^+^CD25^low/-^GITR^+^ and CD4^+^CD25^high^GITR^−^ cells from HC over heterologous effectors from SLE patients were also tested (Figure 7A). In this heterologous suppression assay, the regulatory activity exerted by CD4^+^CD25^low/-^GITR^+^ cells from SLE patients resulted higher than CD4^+^CD25^high^GITR^−^ cells from SLE patients as already demonstrated in autologous suppression tests (Figure 5). Data deriving from all patients and HCs is summarized in Figure 8.

Comparison between Figure 8D and C suggests that SLE CD4^+^CD25^low/-^GITR^+^ cells over HC effectors had a much higher regulatory activity than did HC CD4^+^CD25^low/-^GITR^+^ cells over SLE effectors (*P* <0.01), and comparison between Figure 8A and C suggests that HC CD4^+^CD25^low/-^GITR^+^ cells over HC effectors had a higher regulatory activity than over SLE effectors, in accordance with the partial resistance to suppression of SLE effectors [5,6]. Although some studies suggest that the regulatory activity is more active toward autologous effectors, the comparison between Figure 8D and B suggests that SLE CD4^+^CD25^low/-^GITR^+^ cells had a slightly higher regulatory activity over HC effectors than SLE effectors, again confirming the partial resistance to suppression of SLE effectors. Nevertheless, SLE CD4^+^CD25^low/-^GITR^+^ cells are more active than HC ones in autologous suppression assay (Figure 8B and A).

We next compared the effect of CD4^+^CD25^low/-^GITR^+^ cells from HC and SLE patients on the same effector cells from one HC. Figure 7C shows two representative experiments of six. The mean inhibition by CD4^+^CD25^low/-^GITR^+^ cells from HC was equal to 16.8% ±12.9% and the mean inhibition by CD4^+^CD25^low/-^GITR^+^ cells from SLE patients was equal to 27.2% ±14.5%, confirming that the regulatory activity of cells from SLE was higher than that from HC, when the inhibition of the same effectors was evaluated.

To evaluate whether the higher regulatory activity of CD4^+^CD25^low/-^GITR^+^ cells from SLE patients depended on the number of CD4^+^CD25^low/-^GITR^+^ cells, we correlated the percentage of CD4^+^CD25^low/-^GITR^+^ cells in CD4^+^ cells from HC and SLE patients with the inhibition of proliferation of effectors from the same subjects by CD4^+^CD25^low/-^GITR^+^ cells (Figure 7D). Results show that an apparent correlation between the high percentage of CD4^+^CD25^low/-^GITR^+^ cells in CD4^+^ in SLE patients and the high level of regulatory activity of CD4^+^CD25^low/-^GITR^+^ cells from SLE patients is present. However, correlation is not significant, possibly because of the few data analyzed. Surprisingly, an inverse correlation (nonsignificant) was observed in HC.

When comparing regulatory activity of CD4^+^CD25^low/-^GITR^+^ and CD4^+^CD25^high^GITR^−^ cells from HC, the regulatory activity of CD4^+^CD25^high^GITR^−^ was higher than CD4^+^CD25^low/-^GITR^+^ in 18 of 19 assays (Figures 5A, 7A, and 8E). On the contrary, in SLE patients, the regulatory activity was always lower in CD4^+^CD25^high^GITR^−^ cells than in CD4^+^CD25^low/-^GITR^+^ cells (Figures 5B, 7B, and 8E). The difference in the regulatory activity of the two subsets in HC and SLE subject was highly significant, confirming functional differences between SLE and HC subsets, likely due to CD4^+^CD25^high^GITR^−^ cells.

## Discussion

It is recognized that the fine balance between Tregs and effector T cells, regulating immune homeostasis, is often disrupted in autoimmune disorders [1-4]. In particular, some studies demonstrated a decreased activity of Treg in SLE patients [8-11] and, in this setting, we investigated whether the newly described CD4^+^CD25^low/-^GITR^+^ Treg subset is defective in SLE patients. We found that CD4^+^CD25^low/-^GITR^+^ cells display all the properties of Tregs. Interestingly, the mean regulatory activity of CD4^+^CD25^low/-^GITR^+^ cells was higher in SLE patients (though not significantly different) than in HCs. Taking into account that CD4^+^CD25^low/-^GITR^+^ cells in SLE are in greater number than in HCs, even their regulatory activity may appear stronger, accordingly (see Figure 7D). Therefore, the levels of regulatory activity of CD4^+^CD25^low/-^GITR^+^ cell on a cellular basis may be higher, equal to, or even lower in SLE patients than in HCs. The levels of expression of Treg markers in CD4^+^CD25^low/-^GITR^+^ cells were equal or significantly greater in SLE patients than in HCs, suggesting that CD4^+^CD25^low/-^GITR^+^ cells represent activated Tregs [48,49]. The hypothesis is in line with the possibility that effector T cells are partly activated in autoimmune diseases [5,6], thus eliciting Treg activation. In summary, our data overall demonstrate that CD4^+^CD25^low/-^GITR^+^ cells from SLE patients show a Treg phenotype (possibly those of activated Treg) and exert a clear regulatory activity at levels similar to those of HC cells.

After their discovery, CD4^+^CD25^low/-^GITR^+^ subset in HCs were referred to as CD25^low^, despite formally being CD25^−^, as assessed by flow cytometry. CD25^low^ means that their CD25 expression level was higher than in effector cells, as shown by both real-time PCR and flow cytometry [36]. These data were confirmed also in cells from SLE patients, as shown in Figure 1. Moreover, these cells, now referred to as CD4^+^CD25^low/-^GITR^+^, are FoxP3^+^ and CTLA-4^+^, suggesting that in SLE disease they continue to show a regulatory phenotype. In fact, our results confirm an active role of CD4^+^CD25^low/-^GITR^+^ in T-cell suppression because, as observed in HCs, SLE CD4^+^CD25^low/-^GITR^+^ cells produce high amounts of TGF-β and IL-10 and their neutralization, by blocking Abs, inhibits their suppressive activity. Our previous study did not report a role of IL-10 in mediating CD4^+^CD25^low/-^GITR^+^ suppressive activity [36], probably as a consequence of the use of anti-IL-10 neutralizing Ab not tested for functional assays.

We also demonstrated that CD4^+^CD25^low/-^GITR^+^ cells are expanded in SLE patients and show a predominant memory phenotype. Results from our and previous studies strongly suggest that CD4^+^CD25^low/-^GITR^+^ cells are more likely pTregs, such as Tr1, rather than thymic Treg (tTreg). Indeed, they are expanded memory cells and do not suppress effector cells by cell contact but only by production of immunomodulatory cytokines.

Expansion of CD4^+^CD25^low/-^GITR^+^ recalls three recent studies describing the expansion of CD4^+^CD25^−^FoxP3^+^ cell subset in SLE patients [14,22,50]. Because CD4^+^CD25^−^FoxP3^+^ cell subpopulation includes CD4^+^CD25^low/-^GITR^+^ cells, we can suppose that the described expansion of the CD4^+^CD25^−^FoxP3^+^ cell subset is due, at least in part, to the expansion of CD4^+^CD25^low/-^GITR^+^ cells. Notably, Bonelli *et al*. and this study demonstrate that these subsets have regulatory activity [14], whereas Zhang *et al.* [22] do not confirm these findings. As suggested by Horwitz [51], these differences may depend on the evaluated patient. Indeed, Zhang *et al*. studied untreated new-onset SLE patients whereas Bonelli *et al.* and we studied patients with long-standing disease.

The idea that autoimmune diseases derive from functional or numeric imbalance between autoreactive T cells and Tregs is well established. It has been demonstrated that the CD4^+^CD25^high^ cells are less active and/or less frequent in SLE patients [7-11], as confirmed by the present study. Nevertheless, Tregs contribute, at least in part, to the control of the disease, as demonstrated by unmasking autoreactive CD4^+^ T cells with the removal of Tregs [52]. In this setting, it has been hypothesized that subsets of Tregs may arise under specific autoimmune conditions as an effort to counteract the activity of effectors leading to autoimmunity [15]. This hypothesis seems to be confirmed by recent studies describing the expansion of Treg subsets in SLE [12,14,16,50]. Interestingly, we observed an expansion of circulating CD4^+^CD25^low/-^GITR^+^ Treg cells and a correlation between their number and inactive disease, suggesting that expansion of CD4^+^CD25^low/-^GITR^+^ cells is a homeostatic event to control the disease. The findings we recently described in patients with primary Sjӧgren syndrome appears to support this hypothesis [39]. In this context, the results of recent studies showing Treg subset expansion in SLE patients with active disease are only apparently in contrast with our data. The expansion of CD4^+^CD25^−^FoxP3^+^ cells found by Bonelli *et al*. [14] in active SLE was associated with defective Treg function of these cells, thereby suggesting a possible contamination with non-Treg cells [14]. Similarly, the Treg cells identified as CD45RA^+^FoxP3^low^ by Pan *et al*. [13] were expanded in active SLE but with a defective suppressive activity [13]. Finally, the *in vitro* evaluation of regulatory activity of FoxP3^+^Helios^+^ Treg cells, increased in SLE and positively correlated to disease activity, was not feasible for technical problems [16].

When transferring the concept of Tregs from rodents to humans, an important issue was the inability to easily identify human functional Tregs in patients with various autoimmune diseases. For example, it is known that only a subset of T cells identified as CD4^+^CD25^high^ by flow cytometry are functionally suppressive Tregs, whereas others are activated effector T cells [35,53]. In SLE patients, the contamination of CD25^high^ subset with a more activated/autoreactive cells is confirmed by both phenotypic and functional studies. In fact, CD25^high^ cells expressing FoxP3 are in a lower percentage than in HCs [34]. Moreover, CD25^high^ cells display an activated phenotype [5,10] and have a decreased regulatory activity [8-10]. Our study confirms that the regulatory potential of CD4^+^CD25^high^GITR^−^ cells (the most represented among CD25^high^ cells [36]) is weaker in SLE patients than in HCs and that CD4^+^CD25^high^GITR^−^ cells, expressing low levels of Treg markers, are activated cells. Conversely, CD4^+^CD25^low/-^GITR^+^ cells maintain all the phenotypic and functional features of Treg cells. Interestingly, in patients where the percentage of CD4^+^CD25^low/-^GITR^+^ cells is high, the percentage of CD4^+^CD25^high^GITR^−^ cells is low and *vice versa* (Figure 1F). If we consider the majority of CD4^+^CD25^high^GITR^−^ cells as activated effector cells, the inverse correlation may suggest that CD4^+^CD25^low/-^GITR^+^ cells counter expansion of autoreactive/activated cells, as also confirmed by the clinical correlation between CD4^+^CD25^low/-^GITR^+^ cell numbers and disease activity.

It has been hypothesized that an increased number of fully active Tregs could revert the imbalance of suppressor/effector cell ratio and may change the natural history of autoimmune diseases. Indeed, therapies that increase Treg numbers and activity have been shown to be effective at reversing autoimmune diseases in animal models such as experimental autoimmune encephalitis [54] and diabetes [55]. Of note, *ex vivo* expanded regulatory T cells, adoptively transferred in lupus-prone mice, reduced the rate of renal disease development, and a second transfer, in animals that already developed proteinuria, further delayed the progression of renal disease and significantly improved survival [56]. Moreover, a single transfer of TGF-β-conditioned T cells to animals decreased circulating anti-dsDNA Abs, reduced proteinuria, and doubled survival [57]. A phase I/II trial using Tr1 cell clones in patients displaying severe Crohn disease, yielded encouraging results [58] and a dose-escalation trial using autologous *ex vivo* expanded polyclonal Tregs (CD4^+^CD25^+^CD127^-/lo^) in diabetes patients is currently ongoing [59].

We believe that *in vitro* expanded autologous/heterologous Tregs may help treat autoimmune diseases, including SLE, and that CD4^+^CD25^low/-^GITR^+^ cells are interesting from this point of view, because they appear to be a homeostatic attempt to counteract the disease. Protocols to expand CD4^+^CD25^low/-^GITR^+^ cells are under study and may include TCR/GITR co-triggering or TCR/CD28 co-triggering in the presence of immunosuppressive drugs.

## Conclusions

In conclusion, the present data, together with those previously published on patients with primary Sjӧgren syndrome [39], suggest that CD4^+^CD25^low/-^GITR^+^ cells are expanded pTregs in response to an effector/Treg imbalance and are able to control, at least in part, the disease. In SLE patients, the CD4^+^CD25^low/-^GITR^+^ subset does not appear to be contaminated by activated cells devoid of regulatory activity, suggesting that GITR could be used as valuable surface marker of Tregs. Strategies aimed to expand the CD4^+^CD25^low/-^GITR^+^ subset for therapeutic purposes deserve to be investigated.

## Abbreviations

FoxP3: Forkhead box protein P3
HC: healthy control
HPRT: hypoxanthine phosphoribosyl transferase 1
PB: peripheral blood
SLE: systemic lupus erythematosus
SLEDAI: SLE disease activity index
Treg: regulatory T cells
